# The power of saliva: Antimicrobial and beyond

**DOI:** 10.1371/journal.ppat.1008058

**Published:** 2019-11-14

**Authors:** Taissa Vila, Alexandra M. Rizk, Ahmed S. Sultan, Mary Ann Jabra-Rizk

**Affiliations:** 1 Department of Oncology and Diagnostic Sciences, Dental School, University of Maryland, Baltimore, United States of America; 2 Department of Microbiology and Immunology, School of Medicine, University of Maryland, Baltimore, United States of America; Geisel School of Medicine at Dartmouth, UNITED STATES

## Gatekeeper of the oral cavity

**Fig 1:**
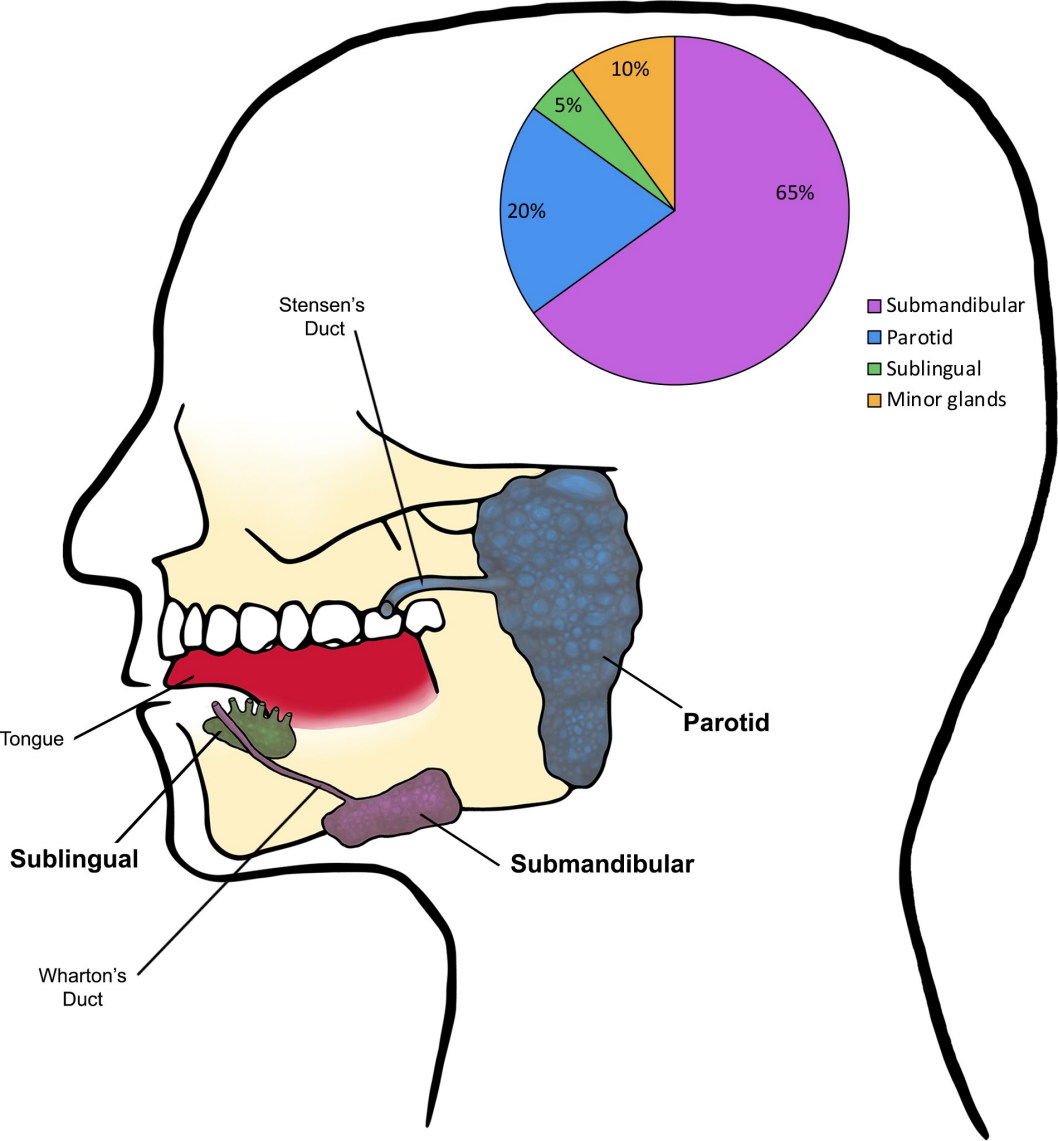
Anatomical location of human major salivary glands and their salivary contribution. Minor salivary glands (not shown) also contribute approximately 10% of total salivary secretions. Salivary contributions in the pie chart are shown based on the resting state; differences in salivary contribution/gland may be observed when saliva is analyzed from stimulated states.

**Fig 2:**
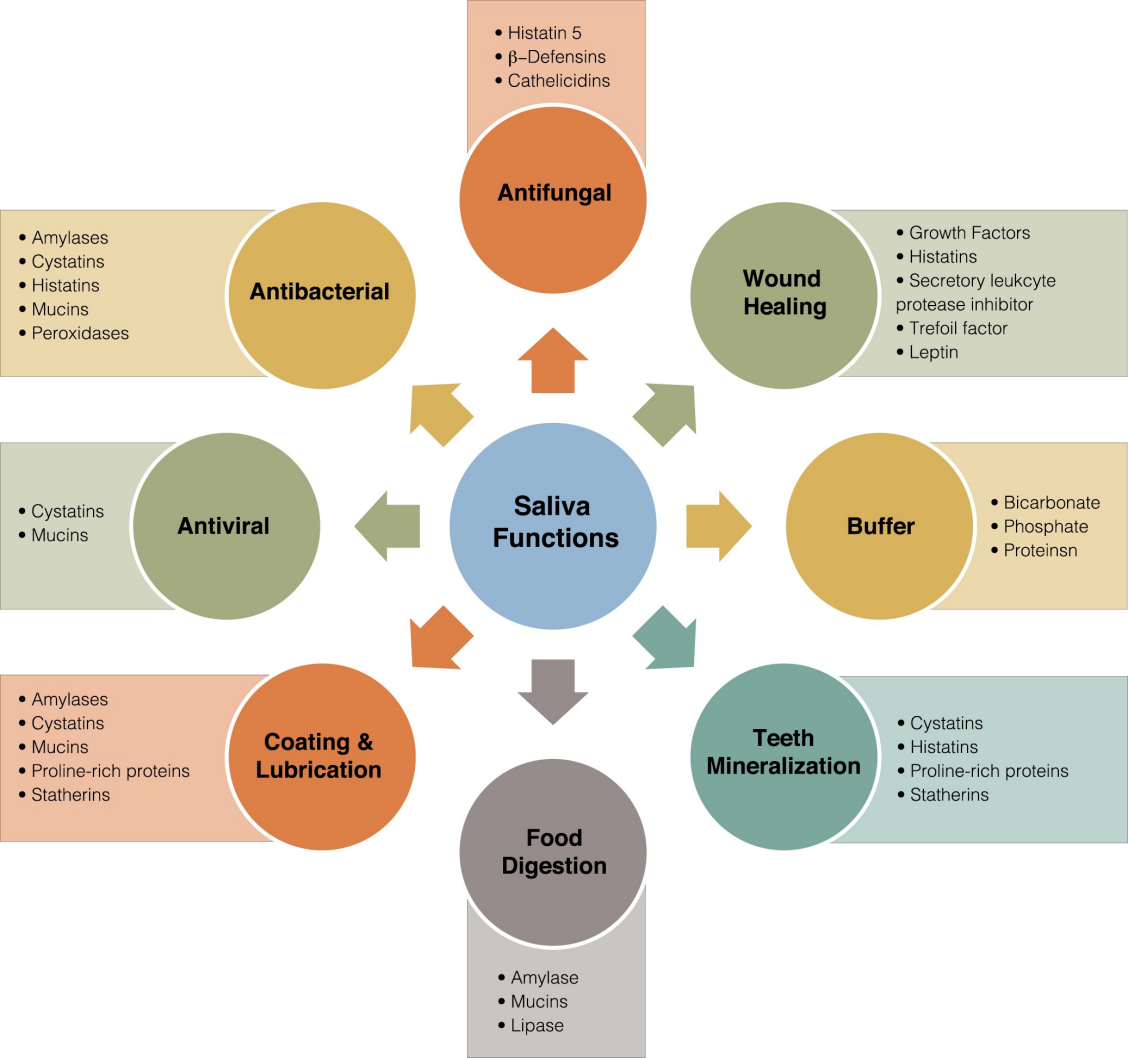
Saliva components and functions.

The oral cavity is a major portal of entry for pathogens that may lead to changes in the normal microflora [1]. The homeostasis of the oral cavity is maintained by saliva, an extracellular fluid produced by salivary glands and secreted in the mouth through openings called salivary ducts [2]. There are three pairs of large salivary glands and hundreds of small (minor) glands; the main glands are the parotid (located in front of and just below each ear), the submandibular (located below the jaw), and the sublingual glands (located under the tongue) (Fig 1). Human saliva is 99% water, and it is estimated that a healthy person produces 600 mL per day; however, during sleep, the amount drops to nearly 0 [3, 4]. Saliva fulfills key functions in the mouth, including maintenance of oral hygiene, lubrication, chewing, and swallowing of food (Fig 2). Additionally, saliva contains several important enzymes such as amylase, lysozyme, and lipase, and therefore, the process of digestion starts the moment food enters the mouth [4]. Importantly, saliva is crucial for defense against microbial species, as it is rich in antimicrobial compounds such as hydrogen peroxide, lactoferrin, and lysozymes [3, 5]. Consequently—in addition to affecting taste, chewing, and swallowing—disruptions in saliva secretion increase the frequency of oral conditions such as oral candidiasis, gum disease, and tooth decay (caries), as well as respiratory tract infections [4, 6]. Xerostomia (a subjective feeling of oral dryness) and salivary hypofunction (a clinically objective decrease in saliva production) are the most commonly reported side effects of medications; however, HIV-AIDS and autoimmune disorders can also lead to salivary dysfunction [2]. Additionally, although infrequent, stones (sialoliths) in the gland ducts may obstruct the flow of saliva, and infections or tumors in the salivary glands may also impact salivary function. Importantly, individuals with all these conditions tend to be highly predisposed to oral candidiasis, likely due to compromised salivary antimicrobial effectors [6]. Therefore, it has become evident that saliva amasses an infinite wealth of beneficial protective and healing properties, particularly in its defense against microbial inhabitants of the oral cavity, commensals and pathogens alike.

## Microbial curator

The warm, nutrient-rich, and moist environment in the oral cavity promotes the growth of a diverse microflora, including resident and transient bacteria and fungi [1]. Saliva plays a dual role in modulating microbial attachment and colonization in the mouth. Dental enamel is coated with a film of salivary pellicles and proline-rich proteins, which bacteria (such as *Streptococcus gordonii*, *Actinomyces naeslundii*, and *Porphyromonas gingivalis* and yeasts) exploit as receptors to adhere to dental surfaces and avoid elimination [7]. On the other hand, the binding of microorganisms to similar receptors in the soluble-phase saliva enhances microbial clearance by promoting agglutination and blocking of surface adhesins [7]. It is well accepted that saliva functions in the manipulation of colonization and limiting attachment of microorganisms to oral tissues; however, the importance of saliva as a component of host immunity is underappreciated. In fact, saliva is rich with effectors that exert direct antimicrobial activity, such as enzymatic breakdown of bacterial cell walls by lysozyme and sequestering iron by lactoferrin [4, 5]. However, the most consequential salivary components involved in defense against microbial species are antimicrobial peptides, which play a vital role in innate immunity as they often constitute the first line of defense against microbial invaders [5, 8]. Due to their positive charge, the general mode of action of these peptides involves binding to the negatively charged surface of microbial membranes, forming pores that ultimately result in lethal efflux of vital cell constituents [9, 10]. Among the best characterized antimicrobial peptides in saliva are the defensins, cathelicidins, and histatins; these diverse peptides can also interact synergistically in limiting microbial colonization [11]. Human β-defensins are a family of evolutionarily conserved cationic peptides mostly produced by epithelial and immune cell populations [12]. These multifunctional peptides mediate the cross-talk between host and microbes and, therefore, play a key role in maintaining a healthy and dynamic equilibrium across the oral mucosal system [13]. The cathelicidins are another group of broad-spectrum antimicrobial peptides derived from both neutrophils and salivary glands that play multiple vital roles in wound healing, immunomodulation, and angiogenesis [2, 11]. The histatins however, a family of 12 histidine-rich cationic peptides, are unique in that they are exclusively produced by the salivary glands of humans and upper primates [14]. Although the histatins exhibit broad-spectrum antimicrobial activity against a wide range of oral microorganisms, histatin-5, specifically, possesses potent antifungal activity, primarily against the fungal pathogen *Candida albicans* [15]. Although the mechanism of action of histatin-5 on the fungal cell is likely multifactorial, it was shown that the process includes binding of histatin-5 to specific receptors on the fungal cell wall and subsequent intracellular uptake in which it targets the mitochondria, ultimately disrupting cell homeostasis [16, 17]. In addition to histatin-5, statherin is another salivary peptide secreted by parotid and submandibular glands with important antifungal activity. Statherin has been shown to, under certain conditions, induce *C*. *albicans* hyphae-to-yeast transition, which could have a protective effect on the oral mucosa by keeping *C*. *albicans* in the commensal yeast form [18].

## Saliva and oral candidiasis

*C*. *albicans* is a pleomorphic fungal species that grows as globular yeasts in its commensal form but can switch to an invasive filamentous form, able to penetrate tissues and cause a variety of mucosal diseases such as oral candidiasis or thrush [19]. Saliva secretion is important for maintenance of the commensal state of *C*. *albicans* in the mouth as it is highly enriched in antifungal proteins such as histatin-5, which helps in limiting *C*. *albicans* attachment to the oral epithelia [20]. Therefore, it is not surprising that salivary hypofunction, characterized as reduced or absent saliva flow, predisposes to candidiasis. Salivary hypofunction, as a consequence of chemoradiation, is a common iatrogenic treatment-associated sequela in head-and-neck cancer patients [6]. However, salivary hypofunction is also considered a hallmark of Sjögren’s syndrome (SS), a chronic inflammatory autoimmune disorder that affects 1–4 million people in the United States, in which immune cells attack and destroy salivary glands [21]. Conversely, SS patients with oral candidiasis also showed elevated levels of salivary calprotectin, another antimicrobial peptide, possibly due to mucosal transudation from inflamed mucosa [22]. Additionally, the function of salivary glands is also reported to be affected by HIV infection [23]. Interestingly, although the association is yet to be fully explored, a clinical study evaluating histatin-5 production demonstrated significantly reduced levels of the peptide in saliva from HIV^+^ individuals, which was concomitant with enhanced candidal colonization [24]. Similarly, patients with hyper-IgE syndrome, a rare congenital immunodeficiency that leads to dramatic Th17 deficits were also shown to have impairment in the production of antimicrobial peptides, including β-defensin 2 and salivary histatins [25]. Although vastly different in disease etiology, patients with these differing salivary disorders are known to be highly predisposed to oral candidiasis, partly due to impaired host defenses, which—in addition to impaired levels of salivary antimicrobial peptides—may include loss of innate immune cell function and/or recruitment [5, 26].

## Beyond

Although the oral cavity is subject to numerous injuries that result in wounds or ulcerations—such as biting of the tongue and cheeks, trauma from dental procedures, or accidental injuries—it is known that wounds of the oral cavity generally heal much quicker than skin injuries [27]. This profound healing capacity of the oral cavity is mainly attributed to the wound healing properties of saliva and specifically to the naturally occurring growth factors that play the most significant role in the wound healing process [28]. The main growth factors found in saliva with reported wound healing properties include epidermal growth factor (EGF), vascular endothelial growth factor (VEGF), transforming growth factor alpha (TFG-α), transforming growth factor beta (TFG-β), nerve growth factor (NGF), fibroblast growth factor (FGF), and insulin-like growth factor (IGF) [4, 27]. Moreover, many additional factors contribute to the regeneration of oral wounds, such as secretory leukocyte protease inhibitor (SLPI)—which prevents the degradation of key proteins involved in connective tissue repair—and trefoil factor 3 (TFF3), which increases the migration of oral epithelial cells [4]. Interestingly, in addition to their antimicrobial activity, the histatins were also shown to possess wound healing properties by enhancing the reepithelialization process and promoting endothelial cell adhesion [28, 29]. Remarkably, opiorphin, a potent natural pain killing substance, is also present in saliva; this endogenous compound, first isolated from human saliva, prolongs the body's own defenses by preventing the breakdown of chemicals that activate opiate receptors that block pain signals from reaching the brain [30].

## Salivaomics: Biomarkers and molecular signatures

Traditionally, the screening, diagnosis, and prognostication of many diseases has relied on using blood samples. However, human saliva is a rich reservoir of different proteins and peptides, and in recent years, it has become evident that salivary constituents become detectably altered in response to certain disease states. Due to recent advances in molecular biology, over 100 molecules detected in salivary samples are evaluated as potential diagnostic or prognostic biomarkers for a variety of conditions, including dental caries, periodontal disease, cancer, diabetes, and many other systemic disorders [3]. In fact, an initiative by the National Institute of Dental and Craniofacial Research has created a roadmap to use oral fluids as a diagnostic medium for scrutinizing the health and disease status of patients [31]. Introduced in 2008, the term "Salivaomics" aimed to highlight the rapid development of knowledge about various "omics" constituents of saliva, including proteome, transcriptome, metabolome, and microbiome [32]. By integrating these technologies in saliva analysis, salivaomics represents a novel approach in oral disease diagnosis, prognosis, and monitoring [33]. The design of reliable biosensors for measuring the salivary levels of metabolic compounds, microorganisms, oncologic markers, and drugs has opened new frontiers for the study of host–pathogen interactions and clinical salivary diagnostics [34]. Importantly, as a diagnostic medium, saliva offers several advantages because its collection, sampling, and processing is fast, inexpensive, and noninvasive; and thus, this oral biofluid has the potential to replace blood in the screening, diagnosis, and prognosis of diseases [3]. As this field continues to develop, some of the challenges that will need to be addressed include the correlation between levels of markers in saliva and serum, the effect of salivary flow rate and stimulation on the concentration of salivary markers, and the effect of proteolytic enzymes derived from the host and oral microorganisms on the stability of certain diagnostic markers. Additionally, certain systemic disorders, numerous medications, and chemoradiation may affect salivary gland function and consequently the volume and composition of saliva, which ultimately may influence the diagnostic usefulness of many salivary constituents [31].

## Concluding remarks

The impressive repertoire of the diverse functions for saliva clearly lay bare the alacrity of this oral biofluid to go above and beyond the call of “antimicrobial” duty. Importantly, the underappreciated wealth of saliva’s beneficial and healing properties on the oral mucosa reverberate the true benefit of our host’s secretions.
